# Circulating neutrophil extracellular trap-forming neutrophils in rheumatoid arthritis exacerbation are majority dual endothelin-1/signal peptide receptor+ subtype

**DOI:** 10.1093/cei/uxae072

**Published:** 2024-08-07

**Authors:** Andrew L Cross, Helen L Wright, Jacqueline Choi, Steven W Edwards, Nelson Ruiz-Opazo, Victoria L M Herrera

**Affiliations:** Institute of Life Course and Medical Sciences, University of Liverpool, Liverpool, UK; Institute of Life Course and Medical Sciences, University of Liverpool, Liverpool, UK; Whitaker Cardiovascular Institute and Department of Medicine, Boston University Chobanian and Avedisian School of Medicine, Boston, MA, USA; Institute of Infection, Veterinary and Ecological Sciences, University of Liverpool, Liverpool, UK; Whitaker Cardiovascular Institute and Department of Medicine, Boston University Chobanian and Avedisian School of Medicine, Boston, MA, USA; Whitaker Cardiovascular Institute and Department of Medicine, Boston University Chobanian and Avedisian School of Medicine, Boston, MA, USA

**Keywords:** rheumatoid arthritis, DEspR, NETs, neutrophil subsets

## Abstract

Neutrophil extracellular traps (NETs) are associated with rheumatoid arthritis pathogenesis and severity. Since homeostatic NET-forming neutrophils [NET+Ns] have beneficial roles in defense against pathogens, their distinction from pro-injury [NET+N] subtypes is important, especially if they are to be therapeutically targeted. Having identified circulating, pro-injury DEspR+CD11b+[NET+Ns] in patients with neutrophilic secondary tissue injury, we determined whether DEspR+[NET+Ns] are present in rheumatoid arthritis (RA) flares. Whole blood samples of patients with RA flares on maintenance therapy (*n* = 6) were analyzed by flow cytometry (FCM) and immunofluorescence cytology followed by semi-automated quantitative confocal microscopy (qIFC). We assessed clinical parameters, levels of neutrophils and [NET+Ns], and plasma S100A8/A9. qIFC detected circulating DEspR+CD11b+neutrophils and [NET+Ns] in RA-flare patients but not healthy controls. DEspR+[NET+Ns] were positive for citrullinated histone H3 (citH3+), extruded DNA, decondensed but recognizable polymorphic nuclei, and [NET+N] doublet interactions in mostly non-ruptured NET-forming neutrophils. Circulating DNA+/DEspR+/CD11b+/citH3+microvesicles (netMVs) were observed. FCM detected increased %DEspR+CD11b+neutrophils and DEspR+ cell–cell doublets whose levels trended with DAS28 scores, as did plasma S100A8/A9 levels. This study identifies circulating DEspR+/CD11b+neutrophils and [NET+Ns] in RA-flare patients on maintenance therapy. Detection of circulating DEspR+citH3+[NET+Ns] and netMVs indicate a systemic neutrophilic source of citH3-antigen concordant with multi-joint RA pathogenesis. Increased S100A8/A9 alarmin levels are associated with cell injury and released upon NET-formation. As a ligand for TLR4, S100A8/A9 forms a positive feedback loop for TLR4-induced DEspR+neutrophils. These data identify DEspR+neutrophils and [NET+Ns] in RA pathogenesis as a potential biomarker and/or therapeutic target.

## Teaser

Since homeostatic neutrophil extracellular trap (NET)-forming neutrophils [NET+Ns] have beneficial roles in defense against pathogens, their distinction from pro-injury [NET+N] subtypes is important, especially if they are to be therapeutically targeted. Having identified circulating, pro-injury DEspR+CD11b+[NET+Ns] in patients with neutrophilic secondary tissue injury, we determined whether DEspR+[NET+Ns] are present in rheumatoid arthritis (RA) flares. Whole blood samples of patients with RA flares on maintenance therapy (*n* = 6) were analyzed by flow cytometry and immunofluorescence cytology followed by semi-automated quantitative confocal microscopy.

## Graphical Abstract

**Graphical Abstract:**
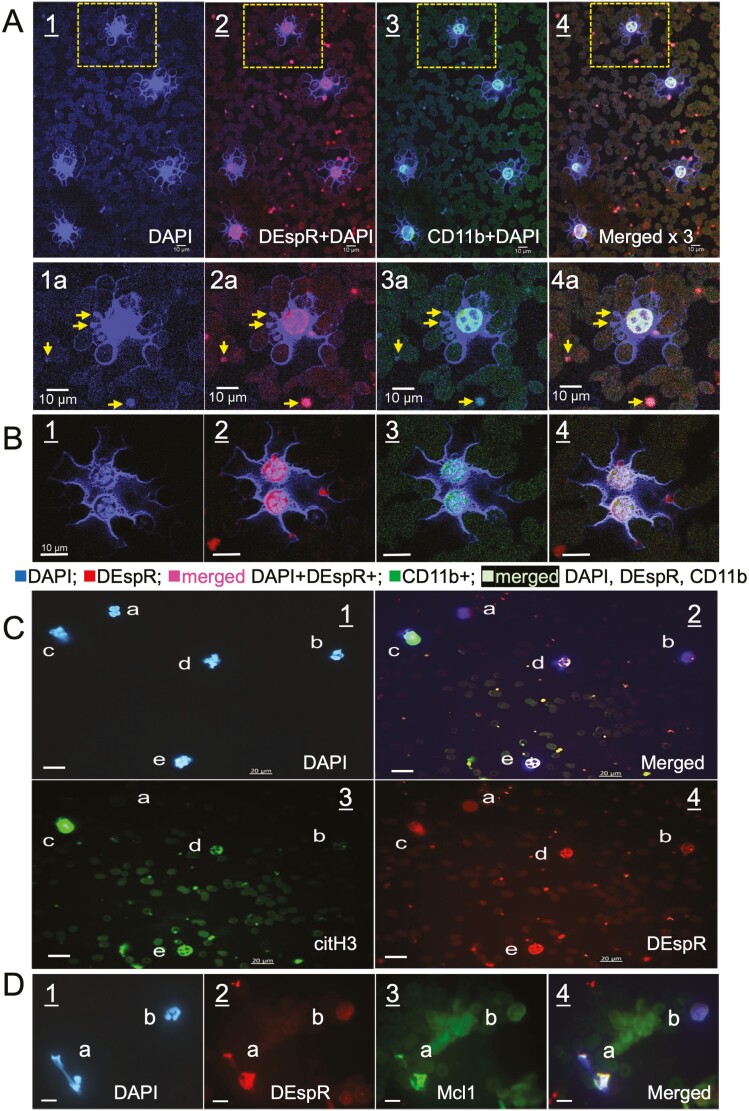

## Introduction

Rheumatoid arthritis (RA) is a chronic autoimmune disorder characterized by inflammation of the synovium, leading to joint damage and systemic complications. Neutrophils play a crucial role in the pathogenesis of RA, contributing to both chronic inflammation and tissue damage [1]. In sterile inflammation, neutrophils are typically the first responders to tissue injury. In RA, the accumulation of neutrophils within synovial joints leads to the pathogenic release of reactive oxygen species (ROS), proteases such as neutrophil elastase and collagenase, and neutrophil extracellular traps (NETs) [1]. The sustained release of ROS, proteases, and an array of chemokines by neutrophils contribute to oxidative stress within the synovial microenvironment, exacerbating inflammation and promoting joint damage [2]. While NETs function in host protection to capture and eliminate pathogens, their release in RA contributes to the chronic inflammation and exposure of antigenic, post-translationally modified peptides leading to tissue damage [3, 4]. In addition, NET-derived neutrophil elastase triggers a vicious cycle of cartilage destruction, fibroblast activation, presentation of antigen to auto-reactive T cells, production of auto-antibody immune complexes and ultimately further neutrophil activation and NET release [3].

Neutrophils also release the calcium-binding proteins S100A8 and S100A9, which act as pro-inflammatory mediators, amplifying the immune response in the synovium. These proteins contribute to the recruitment of other leukocytes, enhancing ROS production, and stimulating the release of inflammatory cytokines [5]. S100A8 may also act as a chemokine, increasing the binding affinity of integrin αMβ2 (Mac-1, CD11b/CD18) on neutrophils and acting as a chemoattractant [6]. Prolonged survival of neutrophils in the inflamed joints of people with RA sustains inflammation and prolongs tissue damage [7].

These observed properties of neutrophils and increased NETs in RA patients align with the properties observed in neutrophils expressing the dual endothelin-1/signal peptide receptor (DEspR). Briefly, DEspR+CD11b+ activated neutrophils exhibit: (a) increased survival compared to DEspR[−] neutrophils [8]; (b) NET-formation while still in the circulation in sterile inflammation [9], and (c) associated with mortality and clinical measures of severity in acute neutrophil-mediated secondary tissue injury such as acute respiratory distress syndrome (ARDS) [9] and spontaneous primary intracerebral hemorrhage (sICH) [10]. Minimal or no circulating DEspR+CD11b+neutrophils are observed in healthy controls, with some exhibiting inducibility *ex vivo* by endotoxin [9]. As subset-specific distinction of neutrophils is key to therapeutically modulating pathogenic neutrophils while sparing homeostatic neutrophil subtypes, we tested the hypothesis that long-lived, pro-injury DEspR+neutrophils capable of NET-formation in the circulation in sterile inflammation are present in RA patients during clinical exacerbation or RA flare, despite pro-neutropenia RA maintenance therapy.

## Materials and methods

### Ethical approval

The study was approved by the NRES Committee North West (Greater Manchester West, Manchester, UK) for the isolation of neutrophils from blood. All participants gave written, informed consent in accordance with the Declaration of Helsinki. All RA patients fulfilled the ACR 2010 criteria for the diagnosis of RA and were Biologics naïve. Six people with RA-exacerbation on standard disease-modifying anti-rheumatic drug (DMARD) therapy were recruited from Liverpool University Hospital Foundation Trust in Liverpool, UK. Patient characteristics are shown in Table 1.

**Table 1:** clinical characteristics of RA patients included in the study

RA patients, *n*	6
Age, mean (range), years	56.4 (44.0–67.9)
Sex, M:F	2:4
ESR, mean (range), mm/h	17.3 (5–31)
CRP, mean (range), mg/L	6.2 (1–19)
DAS28	4.26 (3.55–5.21)
DMARD therapy, *n*	MTX = 3 SSZ = 4 BAR = 1

Abbreviations: ESR: erythrocyte sedimentation rate; CRP: C-reactive protein; DAS28: 28-joint disease activity score; MTX: methotrexate; SSZ: sulfasalazine; BAR: baricitinib.

### Blood collection

Peripheral blood was collected at diagnosis of RA flare by venipuncture into lithium heparin vacutainers before any treatment adjustments. Samples were processed within 1h of blood draw for flow cytometry, plasma, and blood smear preparation for quantitative immunofluorescence cytology (qIFC) with fixation in 100% methanol (−20°C) × 10 min, then stored dry at −20°C as described [9]. The neutrophil-to-lymphocyte ratio (NLR) was calculated from the ratio of the absolute neutrophil count to absolute lymphocyte count measured clinically in the complete blood count differential in peripheral whole blood.

### Flow cytometry

Analysis of patient blood samples was performed by FCM as previously described [10] using AF647-labeled anti-DEspR humanized IgG4 antibody [10] and AF488-labeled anti-CD11b (BioLegend). Forward (FSC) and side scatter (SSC) gating were performed to assess neutrophils, distinguished from monocytes and lymphocytes. RBC lysis was done only after antibody binding and 1% PFA fixation.

### Immunofluorescence staining of cytology slides and analysis

Direct qIFC staining was performed as described [10] in order to optimize cell morphology preservation with fluorophore labeled antibodies: humanized anti-DEspR mab labeled with AF568 paired with AF488-conjugated: (a) CD11b, or (b) citrullinated histone H3 (citH3) (Abcam). Digital confocal (60×-oil) photomicroscopy, or epifluorescence (40×-air, 100×-oil) were performed for analysis of neutrophils, NET-forming neutrophils and neutrophil derived microvesicles. Semi-automated qIFC analysis of NET-forming neutrophils was performed using shape analysis by Nikon BioImaging Lab, as described [9,10].

## Results

### Circulating DEspR+CD11b+ NET-forming neutrophils in RA-flare patients

**Figure 1:**
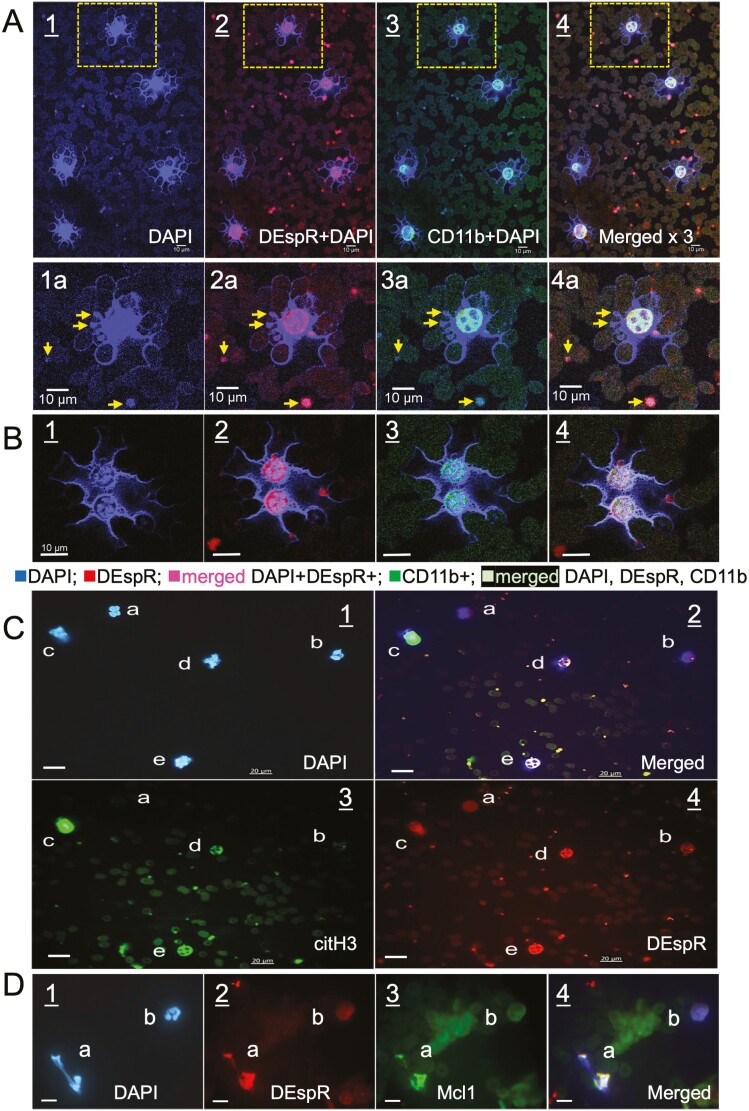
representative confocal-microscopy images of circulating DEspR+[NET+Ns]. (A) DEspR+CD11b+[NET+Ns] showing extruded DAPI+DNA, DEspR+ and CD11b+ (merged). DEspR+CD11b+DAPI+ microvesicles (arrows). Higher magnification images of [NET+N] (box a). (B) Doublets of DEspR+CD11b+[NET+Ns] with extruded DNA and maintained cell morphology. (C) Epifluorescence of [NET+Ns]. Panel-1: non-[NET+N] (a, b), and [NET+Ns] in different stages of NET-formation (c, d, e). Panel-2: [NET+N] (c, d, e) DAPI+DEspR+citH3+; non-[NET+N]: (a, b) citH3-DEspR^LOW^. Panel-3:citH3+[NET+Ns] (c, d, e). Panel-4: DEspR+ immunostaining stronger in [NET+Ns] compared with non-[NET+N]. (D) Representative image of circulating DEspR+Mcl1+ NET-forming neutrophils in RA-flare patient. Panel shows merged image of #a: NET-forming neutrophil, and #b, non-NET-forming neutrophil. DAPI stain shows extruded NET-DNA still attached to a polylobular neutrophil (#a), and a non-NET releasing still polylobulalr neutrophil (#b).

**Figure 2:**
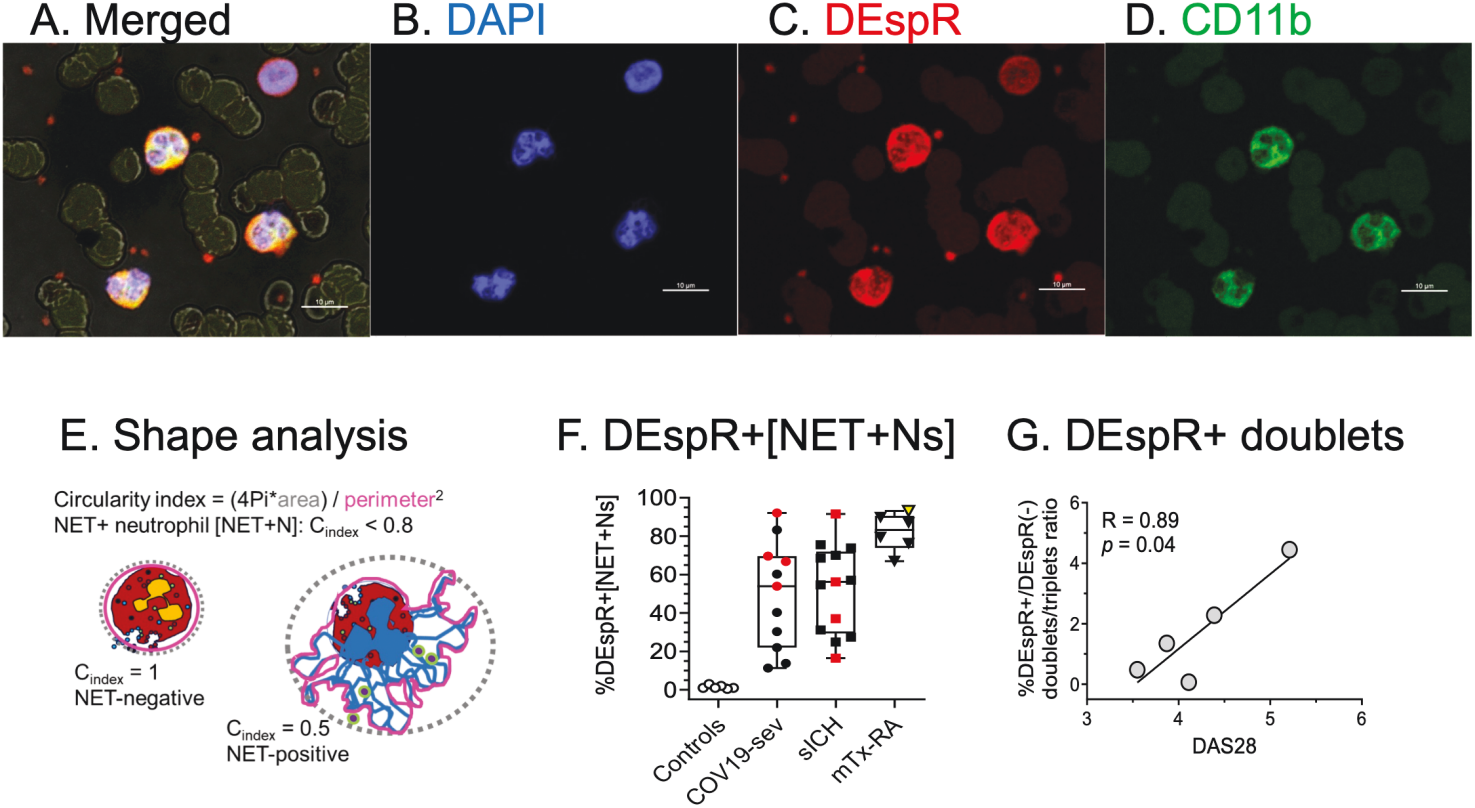
shape analysis of [NET+Ns]. Representative confocal photomicroscopy images of non-NET-forming neutrophils: (A) merged DAPI+, DEspR+ and CD11b+, bright field images; background red blood cells; (B) DAPI+ DNA in polymorphonuclear neutrophils, and band cell (upper right); (C) DEspR+ neutrophils and surrounding microvesicles; (D) CD11b+ neutrophils, band cell is CD11b[−]. Bar = 10 microns. (E) Measurement of [NET+N] via circularity index (*C*_index_): < 0.8 cut-off for NET-formation, non-NET-formation ≥ 0.8. (F) %DEspR+[NET+Ns] in severe COVID-19, spontaneous intracerebral hemorrhagic stroke (sICH), and RA-flare patients (mTx-RA). Red symbols represent patients who died in ICU. Open triangle represents RA patient with highest DAS28 score. (G) Analysis of non-singlets on FCM concordant with [NET+Ns] doublets detected via IFC (Fig. 1B).

Confocal microscopy of qIFC slides detected NET-forming neutrophils [NET+Ns] with extruded DNA in peripheral blood samples (Fig. 1A, panels 1–4) in all RA-flare patients on maintenance therapy in this pilot observational study. Representative high magnification confocal microscopy analysis of peripheral blood cytology slides reveals tentacle-like DNA (Fig. 1A, panel 1) extruded from neutrophils that are non-ruptured and with cell/sub-cell-specific expression of CD11b and DEspR (Fig. 1A, panels 2–4). Observed [NET+Ns] exhibit different levels of nuclear decondensation (Fig. 1A vs. 1B, 1C-1, 1D-1). Higher magnification photomicroscopy images of [NET+Ns] show DAPI+(DNA+) budding from the decondensed nucleus (Fig. 1A, panels 1a–4a), corroborating observations by others of nuclear budding in NET-formation [11], and non-ruptured neutrophil morphology of vital NET-formation-induced *in vitro* with shear [12]. Images also show [NET+N]-derived microvesicles (netMVs) that are DNA+ (DAPI+), DEspR+ and CD11b+ (Fig. 1A-panels 1–4, and panels 1a-d). Images show [NET+N] interactions in whole blood samples: interaction with adjacent red blood cells (RBCs) with DNA wrapped around RBCs appearing as ‘ringlets’ in the images (Fig. 1A and B), and formation of [NET+N] doublets (Fig. 1B). Figure 1D panels 1–4 shows a [NET+N] that is DEspR+Mcl1+ with extruded DAPI+ DNA and still partially connected to the body of the originating neutrophil. Representative photomicroscopy images of non-NET-forming neutrophils from a non-ARDS patient [9] are shown in Fig. 2A–D. This individual was a critically ill patient in the ICU, was not COVID-19 positive, and did not fulfil the Berlin definition for ARDS.

### DEspR+CD11b+ [NET+Ns] and neutrophils in RA-flare patients

Quantitative IFC for NET+Ns using shape analysis algorithms (Fig. 2E) detected that the majority (>60%) of [NET+Ns] are DEspR+CD11b+ in RA-flare patients on maintenance therapy, reaching the high % levels seen in patients with severe COVID-19 [9] and spontaneous intracerebral hemorrhage (sICH) [10] (Fig. 2F). DEspR+CD11b+ [NET+Ns] were not observed in healthy controls and non-ARDS patients [9] (Fig. 2F). Concordant with qIFC-images of doublets (Fig. 1B), flow cytometry detected DEspR+CD11b+ doublets/triplets. Interestingly, the ratio of DEspR+ to DEspR[−] CD11b+ doublets/triplets exhibited a trend to increase with increasing DAS28 score (Fig. 2G), providing basis for further study.

**Figure 3:**
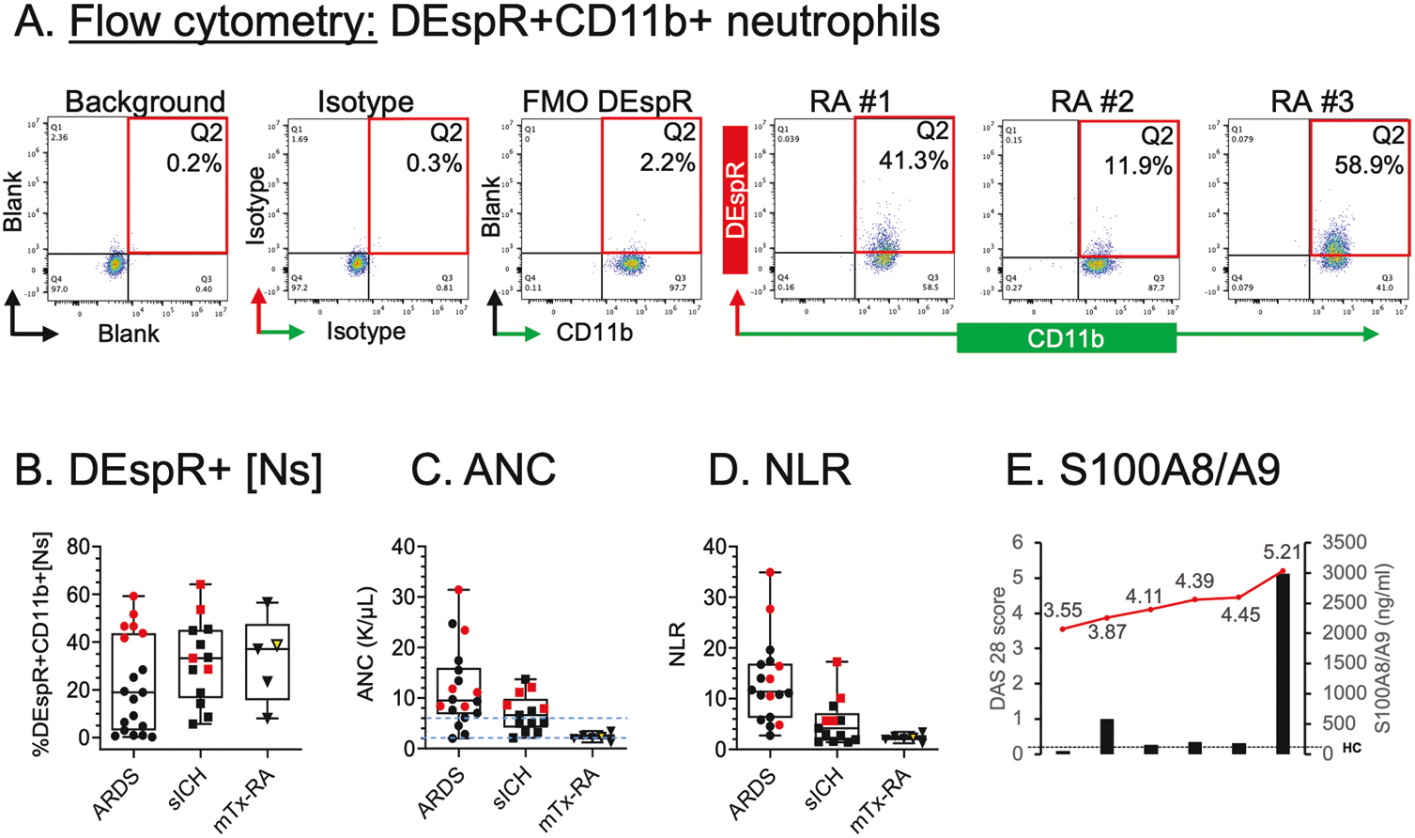
analysis of neutrophil-based markers and S100A8/A9 in RA flare patients. (A) Flow cytometry of circulating DEspR+CD11b+ neutrophils. (B) DEspR+CD11b+[N]-levels (% of all neutrophils) in RA-flare patients (mTx-RA) compared to ARDS and sICH. Analysis of corresponding (C) absolute neutrophil counts (ANC), (D) neutrophil:lymphocyte-ratio (NLR), (E) S100A8/A9 plasma-levels (ng/mL) plotted with corresponding DAS28-scores. Maximum plasma-levels in HC shown by dotted line. Red symbols in panels B, C, D represent patients who died in ICU. Open triangle represents RA patient with highest DAS28 score (>5).

Flow cytometry analysis of circulating DEspR+CD11b+ neutrophils (35 308 ± 17 647 analyzed per subject) and [NET+Ns] (1428 ± 576 analyzed per subject) detected patient-specific levels of %DEspR+CD11b+neutrophils among all neutrophils gated by forward and side scatter properties in RA-flare patients (Fig. 3A), similar to the range of % DEspR+CD11b+neutrophils observed in patients with ARDS and sICH following identical protocols (Fig. 3B). The %DEspR+CD11b+neutrophils in RA patients are notable given that absolute neutrophil counts (Fig. 3C), and NLR (Fig. 3D) are much lower in RA-flare patients compared to ARDS and sICH patients due to standard-of-care RA-maintenance therapy with pro-neutropenia side effects. Notably, plasma levels of S100A8/A9 alarmin are increased in RA flare compared to levels found in healthy controls (mean 59 ng/mL, range 46–107 ng/mL, Fig. 3E), and exhibit a trend showing a marked increase in S100A8/A9 with DAS28 score >5 consistent with reports of association of S100A8/A9 in the highest quartile with severity in RA [13].

## Discussion

The detection of circulating DEspR+CD11b+[NET+N]-subtype in RA-flare patients identifies a long-lived neutrophil-subtype that is trackable and targetable [8] and associated with poor outcomes and NET-formation in the circulation in sterile inflammation in sICH [10]. Concordantly, DEspR’s ligand, endothelin-1, and positive modulator, S100A8/A9 alarmin are both increased in RA [14, 15].

Direct visualization of [NET+Ns] in patient’s whole blood samples revealed that most circulating [NET+Ns] are DEspR+, with extruded DNA but relatively intact and still recognizable polylobulated nuclei albeit in varying stages of decondensation. These observations suggest a non-disruptive release of NETs in the circulation under shear stress that is distinguished from *in vitro* observations of suicidal NETosis [12]. Similarly, the detection of circulating citH3+DNA+ [netMVs] in patients with RA flares identifies a neutrophilic source of citH3+ antigen in RA pathogenesis. The direct visualization of citH3+DNA+ netMVs budding from nuclei in [NET+Ns] presents a potential microvesicle-mediated modality for amplification of circulating citH3 toxicity in RA. Direct visualization of [NET+Ns] provides cell-type pathogenic insights not attained by analysis of citH3 remnants by ELISA or *ex vivo* stimulation of [NET+Ns]. The observed co-expression of Mcl-1 in circulating DEspR+citH3+[NET+Ns] aligns with the pathogenic hypothesis that as Mcl-1 underlies neutrophil extended survival [16], circulating DEspR+Mcl1+non-disrupted [NET+Ns] resist apoptosis-dependent clearance, thus contributing to the association of increased NETs with severity in RA.

Notably, despite low absolute neutrophil counts from standard-of-care RA DMARD therapy, the percent level of DEspR+CD11b+ neutrophils and NET+Ns are relatively high comparable to %levels seen in ARDS [9] and sICH [10]. These observations are concordant with key reports by others on vital or non-rupture neutrophil NET formation [12], long-lived neutrophils and NETs in RA pathogenesis [1], release of microvesicles upon NETs-release [11], and pathogenic roles of NETs in RA pathogenesis [1]. The detection of increased S100A8/A9 alarmin levels with a trend for alignment with DAS28 scores is concordant with a correlation of increased levels of S100A8/A9 with different measures of RA activity and/or severity [15]. As S100A8/A9 alarmins are also released upon NET-formation [17], and activate neutrophil TLR4, whose activation increases DEspR+CD11b+ neutrophils [9], increased S100A8/A9 and increased DEspR+neutrophils in RA comprise a putative self-sustaining cycle of reciprocal interactions.

There are several important limitations to our study. Our sample size was small and did not include follow up data on changes in the population [NET+N] neutrophil subtypes in response to DMARD therapy. We were also not able to include blood samples from DMARD-naïve patients. Nevertheless, we believe our results provide novel insight into disease mechanisms in RA, and the potential for subtyping RA patients in neutrophil-driven disease flare that could be further investigated in a larger, longitudinal cohort study. The presence of [NET+N] in multiple chronic diseases exacerbated in association with increased NLR confirms a common pathogenic mechanism driven by neutrophil-mediated tissue injury associated with long lived neutrophils and NET formation. In addition, the detection of increased circulating [NET+N] beyond the confines of the joint aligns with the systemic multi-joint pathology in RA, and the increased risk for cardiovascular disease wherein neutrophils and NETs are also implicated. The identification of circulating DEspR+CD11b+NET-forming neutrophils in our small, but unequivocal study paves the way toward RA-flare subtyping, subsequent subtype-specific targeted therapies and approaches. Reporting the identification of the DEspR+neutrophil subset with capacity to form and release NETs while still in the circulation and in the presence of relative neutropenia as side effect of DMARD therapy is important and provides insight into why RA-flares may occur despite maintenance therapy.

In conclusion, this index-cohort study identifies DEspR+CD11b+ neutrophils and the [NET+N] subtype in RA-flare patients, distinguished from homeostatic DEspR[−] neutrophil subtypes. As DEspR+CD11b+ neutrophils and [NET+Ns] were previously shown to be associated with neutrophilic secondary tissue injury in ARDS and sICH [9, 10], and targetable by anti-DEspR antibody-induced apoptosis [9], observations in this index study support the basis for further studies along the RA pathogenic spectrum as a potential biomarker and/or therapeutic target.

## Data Availability

The data underlying this article will be shared on reasonable request to the corresponding author.

## Abbreviations:

ACR: American College of Rheumatology
ARDS: acute respiratory distress syndrome
BAR: baricitinib
citH3: citrullinated histone H3
CRP: C-reactive protein
DAS28: 28-joint disease activity score
DEspR: dual endothelin-1/signal peptide receptor
DMARD: disease-modifying anti-rheumatic drug
ESR: erythrocyte sedimentation rate
FCM: flow cytometry
Mcl-1: myeloid cell leukemia differentiation protein 1
MTX: methotrexate
NET: neutrophil extracellular trap
netMVs: circulating DNA+/DEspR+/CD11b+/citH3+ microvesicles
NET+N: NET-forming neutrophils
NLR: neutrophil to lymphocyte ratio
qIFC: quantitative confocal microscopy
RA: rheumatoid arthritis
RBCs: red blood cells
ROS: reactive oxygen species
sIHC: spontaneous primary intracerebral hemorrhage
SSZ: sulfasalazine
TLR: toll-like receptor
